# Histoplasmosis by* Histoplasma capsulatum* var. duboisii Observed at the Laboratory of Pathological Anatomy of Lomé in Togo

**DOI:** 10.1155/2017/2323412

**Published:** 2017-07-18

**Authors:** Tchin Darré, Bayaki Saka, Abas Mouhari-Touré, Améyo Monique Dorkenoo, Koffi Amégbor, Vincent Palokinam Pitche, Gado Napo-Koura

**Affiliations:** ^1^Department of Pathology, University Teaching Hospital of Lomé, Lomé, Togo; ^2^Department of Dermatology, University Teaching Hospital of Lomé, Lomé, Togo; ^3^Department of Parasitology and Mycology, University Teaching Hospital of Lomé, Lomé, Togo

## Abstract

Our study aimed to describe the epidemiological, clinical, and diagnostic aspects of African histoplasmosis in Togo through a descriptive and cross-sectional study on histological diagnosed African histoplasmosis in Pathology Department of Lomé from 2002 to 2016 (15 years). A total of 17 cases of African histoplasmosis were diagnosed. The sex ratio (M/F) was 1.8. The annual incidence was 1.1 cases. The mean age of the patients was 27.2 ± 0.4 years. All our patients were of social categories with a low socioeconomic level. HIV infection was known in 3 patients and one patient contracted tuberculosis. The clinical manifestations were cutaneous in 7 cases, cutaneous and mucous in 3 cases, cutaneous and lymph node in 3 cases, cutaneous and bone in 2 cases, and disseminated in 2 cases. The samples examined consisted of 14 cutaneous biopsies measuring 2-3 cm and 3 ganglionic biopsies each measuring 4 cm of major axis. Histologically, all cases were of chronic form made of granulomatous reaction with ovoid yeasts measuring between 1 and 2 microns. Despite the low frequency of this disease in our country, it should be kept constantly in mind before any granulomatous lesions, especially in the context of the HIV pandemic.

## 1. Introduction

Histoplasmosis is a deep mycosis due to a fungus,* Histoplasma capsulatum*, which possesses two varieties, namely,* Histoplasma capsulatum* var.* Capsulatum*, an agent of the so-called American histoplasmosis (wrongly, because of global distribution), and* Histoplasma capsulatum* var.* duboisii*, an agent of African histoplasmosis [1]. They are rarely described in Africa. Indeed, the actual frequency of the African form is not known, as most studies have focused on clinical cases [2, 3]. In contrast, reports of African histoplasmosis during HIV infection are rare, although the two pathogens coexist in our regions [4, 5]. This study on African histoplasmosis, the first of its kind in our country, aimed to describe the sociodemographic and histological aspects observed at the Laboratory of Pathological Anatomy.

## 2. Materials and Methods

This was a retrospective and descriptive study on all records (records and reviews) of histoplasmosis histologically diagnosed in Pathology Department of University Teaching Hospital of Lomé from January 2002 to December 2016, period of 15 years. During this period, the samples were recorded in the laboratory's laboratory record, prepared in thin sections, paraffin-embedded (56–60°C), and then stained with hematin eosin. We reviewed the results of HIV serology and mycological, macroscopic, and histological aspects of African histoplasmosis lesions.

### 2.1. Statistical Analysis

Statistical processing and analysis of data was performed using SPSS software.

### 2.2. Ethical Consideration

This study received approval from the Head of the laboratory department to be conducted. Since it was counting records, patient consent was not required. However during the counting and data collection patient names were not collected in order to preserve confidentiality.

## 3. Results

### 3.1. Epidemiology

Seventeen cases of African histoplasmosis were diagnosed: 11 men (64.7%) and 6 women (35.3%), one sex ratio (M/F) of 1.8. The annual prevalence was 1.1 cases. The average age of patients in our series was 27.2 ± 0.4 years, with extremes of 11 and 63 years. All our patients were of social categories with a low socioeconomic level: 9 were pupils, 5 farmers, and 3 unemployed. The majority of patients (15/17) were from rural areas and had a relatively stable frequency with 1 to 2 per year. Four patients (4/17) had at least one immunosuppressive factor: HIV infection was known in 3 patients and one patient contracted treated tuberculosis. Clinical manifestations were localized to the skin in 7 cases, cutaneous and mucosal in 3 cases, cutaneous and ganglionic in 3 cases, cutaneous and bone in 2 cases, and disseminated in 2 cases. The epidemiological characteristics of the patients are summarized in Table 1.

### 3.2. Histopathology

Skin lesions were often subcutaneous swelling, resembling a cold abscess evolving towards spontaneous fistulization (*n* = 3 cases), or ulcerations (*n* = 2 cases), and sometimes budding (*n* = 1 case). These cutaneous lesions can extend to give large, ulcerative vegetation closets (*n* = 1 case). The mucous lesions were budding. Direct mycological examination and culture in 8 patients were positive in 5 patents. The samples examined consisted of 14 cutaneous biopsies measuring 2-3 cm and 3 ganglionic biopsies each measuring 4 cm of major axis. Histologically, all cases were chronic. In 11 cases the lesions consisted of a granulomatous reaction of multinucleated giant cells, epithelioid cells, and lymphoplasmocytes with ovoid yeasts. In 6 cases the lesions were fibrocaseous with hyalinization and calcification. The pathologic aspects are summarized in Table 2.

## 4. Discussion

### 4.1. Discussion of Epidemiology

Our study is a retrospective study of African histoplasmosis by* Histoplasma duboisii* histologically confirmed by the Laboratory of Pathological Anatomy of the University Hospital of Lomé over a period of 15 years. This work brought together all histological diagnosed cases in Togo's only pathological anatomy laboratory. Cases have escaped us, for patients who prefer to refer to traditional healers, or who are late in visiting health centers where histological specimens are not taken. In addition, other limitations are found in this retrospective study: the lack of certain demographic data and the lack of microbiological and therapeutic data were the limitations of our study. African histoplasmosis is still a rare disease outside a few foci in some African countries [2, 3]. In Congo, 14 cases in 10 years of African histoplasmosis have been reported, confirming the low incidence of this disease in Africa as well as in other continents [6, 7]. The male predominance observed in our series has been reported by the literature which estimates a sex ratio between 2 and 3 [1, 4, 8]. Most of the cases in our series were observed in young subjects consistent with data from the literature, which states that African histoplasmosis is predominantly observed in children under 15 years of age [4–8]. Of the 17 cases in our series, 3 cases were observed in patients with acquired immunodeficiency syndrome (AIDS) and one case associated with tuberculosis. In Africa, most of the reported cases are prior to the AIDS pandemic. These are very often clinical cases and the actual frequency of histoplasmosis is not known [9, 10]. Recent African literature mentions very few cases of histoplasmosis associated with HIV infection [4]. We have not noticed an increase in the number of cases since the advent of AIDS (1 to 2 per year on average), contrary to cryptococcosis [11].

### 4.2. Discussion of Histopathology

These examined specimens, consisting of cutaneous biopsies and lymph nodes, are related to the clinical manifestations of histoplasmosis, which are often mucocutaneous, ganglionic, and osseous [6, 9]. Although lesions are often localized, disseminated forms have been described in children by other authors associating a generalized, multiple osteoarticular, multiganglionic, hepatic, and splenic cutaneous localization [6, 10]. The polymorphism of cutaneous lesions has also been described by some authors including nodules, lesions of pseudomolluscum contagiosum, flat papules, cold abscesses, and budding ulcerations [4, 7].

The anatomopathological aspects encountered in Togo are quite classical and allow a rather reliable diagnosis approach even without mycological culture. These are the epithelioid and purulent granulomas, with lymphocytes, neutrophils, and many giant cells. The diagnosis has always allowed the detection of ovoid yeasts, typical of histoplasmic duboisii by histological examination [7, 11].

## 5. Conclusion

The actual prevalence of African histoplasmosis is underestimated because of its rarity outside endemic areas, delayed diagnosis (clinical polymorphism causing confusion with tuberculosis), the often rapid death of patients (lack of means, financial constraints, and geographical inaccessibility to hospitals), limited technical facilities in our country, and inaccessibility to HIV testing in rural areas. Despite the low frequency of this disease in our country, however, it should be kept in mind at all granulomatous lesions, particularly in the context of the HIV pandemic.

## Figures and Tables

**Table 1:** Epidemiological characteristics of patients.

Characteristics	Values
*Sex*	
(i) Men	11/17
(ii) Women	6/17
*Age (years)*	
(i) Average	27.2 ± 0.4
(ii) Extremes	11–63
*Profession*	
(i) Unemployed	3/17
(ii) Farmers	5/17
(iii) Pupils	9/17
*Immunosuppressive factor (4/17)*	
(i) VIH	3/4
(ii) Tuberculosis	1/4
*Localization*	
(i) Skin	7/17
(ii) Cutaneous and mucosal	3/17
(iii) Cutaneous and ganglionic	3/17
(iv) Cutaneous and bone	2/17
(v) Disseminated	2/17

**Table 2:** Pathologic characteristics of patients.

Characteristics	Values
*Mycological (8/17)*	
(i) Positive	5/8
(ii) Negative	3/8
*Biopsies examined *	
(i) Cutaneous	14/17
(ii) Ganglionic	3/17
*Histological*	
(i) Granulomatous reaction with ovoid yeasts	11/17
(ii) Hyalinization and calcification ovoid yeasts	6/17
